# Determinants of Diabetes Disease Management, 2011–2019

**DOI:** 10.3390/healthcare9080944

**Published:** 2021-07-26

**Authors:** Lawrence V. Fulton, Omolola E. Adepoju, Diane Dolezel, Tahir Ekin, David Gibbs, Barbara Hewitt, Alexander McLeod, Winston Liaw, Cristian Lieneck, Zo Ramamonjiarivelo, Ram Shanmugam, Lechauncey D. Woodward

**Affiliations:** 1 Department of Health Administration, College of Health Professions, Texas State University, San Marcos, TX 78666, USA; clieneck@txstate.edu (C.L.); zramamonjiarivelo@gmail.com (Z.R.); shanmugam@txstate.edu (R.S.); 2 Humana Integrated Health Sciences Institute, College of Medicine, University of Houston, Houston, TX 77004, USA; oadepoju@central.uh.edu (O.E.A.); wliaw@central.uh.edu (W.L.); lwoodard@central.uh.edu (L.D.W.); 3 Department of Health Information Management, College of Health Professions, Texas State University, San Marcos, TX 78666, USA; dd30@txstate.edu (D.D.); dgibbs@txstate.edu (D.G.); barbarah@txstate.edu (B.H.); am@txstate.edu (A.M.); 4 Department of CIS&QM, McCoy College of Business, Texas State University, San Marcos, TX 78666, USA; tahirekin@txstate.edu

**Keywords:** diabetes, ACA, Medicaid, quasibinomial

## Abstract

This study estimated the effects of Medicaid Expansion, demographics, socioeconomic status (SES), and health status on disease management of diabetes over time. The hypothesis was that the introduction of the ACA and particularly Medicaid Expansion would increase the following dependent variables (all proportions): (1) provider checks of HbA1c, (2) provider checks of feet, (3) provider checks of eyes, (4) patient education, (5) annual physician checks for diabetes, (6) patient self-checks of blood sugar. Data were available from the Behavioral Risk Factor Surveillance System for 2011 to 2019. We filtered the data to include only patients with diagnosed non-gestational diabetes of age 45 or older (*n* = 510,991 cases prior to weighting). Linear splines modeled Medicaid Expansion based on state of residence as well as implementation status. Descriptive time series plots showed no major changes in proportions of the dependent variables over time. Quasibinomial analysis showed that implementation of Medicaid Expansion had a statistically negative effect on patient self-checks of blood sugar (odds ratio = 0.971, *p* < 0.001), a statistically positive effect on physician checks of HbA1c (odds ratio = 1.048, *p* < 0.001), a statistically positive effect on feet checks (odds ratio = 1.021, *p* < 0.001), and no other significant effects. Evidence of demographic, SES, and health status disparities existed for most of the dependent variables. This finding was especially significant for HbA1c checks by providers. Barriers to achieving better diabetic care remain and require innovative policy interventions.

## 1. Introduction

The Affordable Care Act (ACA) was enacted in 2010 to provide affordable health insurance coverage to more United States (U.S.) citizens [1]. According to the United States Census Bureau, over 13% of the population were uninsured in 2013 [2]. The ACA was implemented in 2014 and substantially improved access to health insurance [3], but in 2018, 8.5% of the population remained uninsured [4]. Particularly significant is the increased coverage and subsequent access to care for adults with chronic conditions in the United States [5]. As part of the ACA, the Medicaid Expansion Plan extended coverage to those with incomes below 138% of the poverty level, providing coverage to approximately 40 million U.S. citizens who previously were not covered under other plans [6]. As of 2021, twenty-eight states and Washington D.C. have adopted and implemented Medicaid Expansion, two states (Missouri and Oklahoma) have adopted but not implemented it, and 12 states have done neither [7].

According to the Centers for Disease Control and Prevention (CDC), about half of adults live with at least one chronic disease [8]. Diabetes, one of the most common chronic diseases, affects an estimated 34.2 million adults and is the seventh leading cause of death in the United States [8]. By 2050, over 21% of the U.S. population may struggle with diabetes. While diabetes is not considered curable or reversible, some patients with type 2 diabetes have achieved remission through weight loss [9]. The most typical diabetes progression is hyperlipidemia, hypertension, and impaired fasting glucose, which leads to type 2 diabetes [10]. When left uncontrolled, diabetes often leads to various complications, including nephropathy, retinopathy, neuropathy, and cardiovascular disease [11]. Early identification of high-risk individuals is essential in preventing the development of diabetes, and disease management is critical in preventing or delaying the progression of the disease.

The Centers for Disease Control and Prevention [8] report that 20% of individuals are undiagnosed for diabetes. To successfully manage diabetes, the patient must routinely follow several physiological determinants, including blood glucose monitoring and routine foot checks [12]. Self-management requires education and oversight by healthcare professionals [13]. Glasgow [14] recognized these needs in a diabetes management model, which included three stages: (1) social background and contextual history, continuity of care, outcome management, (2) interaction with providers and self-management activities, and (3) quality of life improvements driven by societal costs. To operationalize a national diabetes prevention program requires a societal level strategic approach [15]. Improved care through insured routine interactions with providers is necessary to maintain diabetic treatment plans [16]. Wagner et al. [17] reported that a high percentage of patients with diabetes lacked healthcare insurance, preventing them from seeking essential diabetic management care, increasing complications, aggravating conditions, and increasing the cost of treatment.

U.S. citizens with diabetes can potentially benefit from health insurance reforms in the ACA through increased coverage, preventive screening and services, prohibitions of lifetime benefit limits, and overall increased focus on the disease through Diabetes Report Cards [6]. Insurance expansion under the ACA demonstrates the potential to improve access to healthcare, increase healthcare utilization, and reduce complications and poor health outcomes for U.S. citizens with diabetes [6]. Additionally, preventive services offered under the ACA may play a critical role in early diabetes diagnosis [18], as evidenced by a recent study that reported increased preventive visits in prediabetic individuals post-ACA [19]. Marino et al. [20] suggested that increases in coverage from the ACA improve access to proper diabetes management and care. After ACA implementation, another study found that Medicaid Expansion positively affected self-reported diabetes management.

Several studies have compared data from pre- and post-ACA. For example, Lee et al. [15] compared patients who were part of the Medicaid Expansion Plan pre-ACA (2011–2013) to post-ACA (2014–2016), but did not find a significant change in care. Marino et al. [20] studied the period 24 months prior to the implementation of the ACA (2012–2013) and 24 months after (2014–2015) and found improvements in post-ACA glycosylated hemoglobin A1c (HbA1c) numbers for Hispanic patients and non-Hispanic black patients who had acquired insurance post-ACA. These prior studies produced mixed results. This study expands the body of knowledge on diabetes management longitudinally and further investigates the impact of demographics, socioeconomic status (SES), health status, and Medicaid Expansion on diabetes management. 

Diabetes management may reduce the complications associated with the disease. The aim of this study is to estimate the effects of the Affordable Care Act (ACA), demographics, SES, and health status on disease management of diabetes over time. In this study, we measure disease management by evaluating the following measures: physician checks of patients’ HbA1c, feet, and eyes; increases in patient education; frequency of doctor visits for diabetes in the past year; self-checks of blood sugar by patients. Based on prior research, we expect that access to health insurance through the ACA will increase diabetic patient disease management [21]. We hypothesize an increase in usage of health care services in the post-ACAC period. Theoretically, patients will address their diabetes through increased blood sugar checks, physician visits, foot checks, diabetic education, eye exams with pupil dilation, and HbA1c checks. 

## 2. Materials and Methods

This research modeled individual predictors of diabetes management over time. We considered changes in the frequency of blood glucose checks, the number of times a healthcare professional tested a patient’s glycated hemoglobin (HbA1c), if a patient has ever taken a class to manage diabetes, how many times in the last year a patient has seen a healthcare professional for their diabetes, time since the patient’s last eye exam dilation, and how many times in the last 12 months a healthcare professional has checked a patient’s feet for irritation or sores. Given other smaller studies, we would expect that improved diabetic coverage and more people being insured would increase diabetic patient care [5,20,21]. 

### 2.1. Data and Software

We analyzed BRFSS data from 2011 through 2019. BRFSS is a national survey designed to gather data about individual health behaviors and has been shown to have moderate reliability [22]. The survey was designed to estimate the population of the United States when complex weights are applied, and various weighting schemes that might be applied [23]. We filtered the data for those individuals 45 and older who had non-gestational diabetes. As complex weights were missing from 393 observations over the 9 years in question, we dropped those negligible observations. From 2011 to 2019, there were 510,991 filtered observations (unweighted) in the final sample. For consistency, we checked variable definitions across all years for consistency in measurement. Data were then recoded and preprocessed, the recodes were made publicly available online [24]. R Statistical Software version 4.03 [25] was used for all computations. The R survey package [26] provided the complex weighting and quasibinomial analysis. Other packages used in the analysis were sourced online [24]. 

### 2.2. Dependent Variables

We used six specific questions to generate the dependent variables and collapsed all variables to binary values. The parentheses below include the BRFSS variables names.

1. About how often do you check your blood for glucose or sugar? (BLDSUGAR).
2. About how many times in the past 12 months have you seen a doctor, nurse, or other health professional for your diabetes? (DOCTDIAB).
3. About how many times in the past 12 months has a health professional checked your feet for any sores or irritations? (FEETCHK).
4. About how many times in the past 12 months has a doctor, nurse, or other health professional checked you for A1C? (CKHEMO3).
5. Have you ever taken a course or class in how to manage your diabetes yourself? (DIABEDU).
6. When was the last time you had an eye exam in which the pupils were dilated, making you temporarily sensitive to bright light? (EYEEXAM1).

### 2.3. Independent Variables

#### 2.3.1. Demographics

As diabetes occurrence is more prevalent with age, we included only age groups 45–54, 55–64, and age over 65 years. Other demographic variables included were race/ethnicity (white non-Hispanic, black non-Hispanic, Hispanic, other race non-Hispanic, multi-racial non-Hispanic, unknown), gender (male, female), and marital status (married, divorced, widowed, separated, never married, unmarried couple, unknown). 

#### 2.3.2. Socioeconomic Status

We mined three SES variables for inclusion in the study: income, education, work status. ‘Current income’ included nine levels between USD 0 and above USD 75,000 as well as ‘unknown.’ ‘Highest level of education’ was a six-level independent variable: less than grade 9, grade 9–11, high school graduate or equivalency, 1–3 years of college, college graduate, unknown. Work status consisted of nine levels: employed for pay, self-employed, out of work greater than or equal to one year but seeking employment, out of work less than one year seeking employment, homemaker, student, retired, unable to work, unknown. 

#### 2.3.3. Health Status

To measure health status, we used health-related variables that were dichotomously coded. These variables were health plan (have/do not have), personal doctor status (have/do not have), annual checkup within one year (yes/no), no money to access doctor due to cost within the last year (yes/no). Another health-related covariate was patient self-assessed general health status (excellent, very good, good, fair, poor, and unknown). 

### 2.4. Medicaid Expansion Linear Splines

For each observation and each year, the state Federal Information Processing Standard (FIPS) code for the resident was used to determine whether Medicaid Expansion was both adopted and implemented in their state of residence [7]. A one-knot linear spline variable [27] was coded 0 for years prior to adoption and implementation and then indexed from 1 to *k* for each year post-implementation. Linear splines serve as a mechanism for tracking pre–post-adoption (a single knot) and can model linearity post-adoption. Equation (1) illustrates the one-knot linear spline formulation for a traditional regression formulation.

(1) $$y=\beta_{0}+\overset{g}{\underset{i=1}{\sum }}\beta_{i}X_{i}+\beta_{g+1}I_{t<k}\left(t+1-k\right)\;I_{t<k}=\{\begin{array}{c} 0,\;t<state\;year\;index\;of\;ACA\;implementation\\ 1,\;t\geq index\;of\;state\;year\;of\;ACA\;implementation \end{array}\;t=\left\{1,\;2,\;\ldots \right\},\;k\leq t$$

In this formulation, the dependent variable (y) becomes a function of the intercept ($\beta_{0}$) plus the non-Medicaid Expansion independent variables ($\overset{g}{\underset{i=1}{\sum }}\beta_{i}X_{i}$). A slope parameter ($\beta_{g+1}$) times an indicator knot for ACA implementation ($I_{t<k}$) times the period since implementation (t+1−k) serves as the Medicaid Expansion independent variable. For periods prior to implementation, the indicator function is zero, and the equation becomes $y=\beta_{0}+\overset{g}{\underset{i=1}{\sum }}\beta_{i}X_{i}$, the traditional regression. For periods at implementation and beyond, the indicator is ‘1,’ and thus the equation becomes $y=\beta_{0}+\beta_{1}\left(t+1-k\right)+\overset{g}{\underset{i=2}{\sum }}\beta_{i}X_{i-1}$, which adds a linear effect for post-implementation. Such a spline formulation effectively models two separate equations, one for pre-implementation and one for post-implementation. 

### 2.5. Missing Data

**Figure 1:**
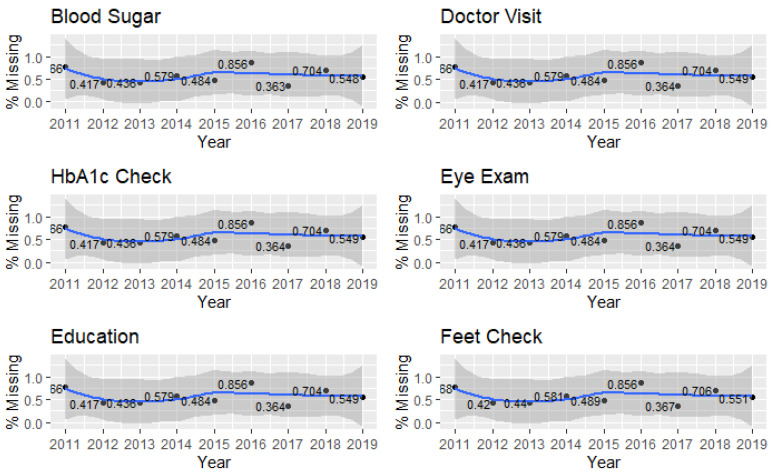
Missing value proportions for each dependent variable by year.

Interviewers for the BRFSS did not ask all respondents all questions, and the states sometimes chose to omit certain questions. Thus, we screened the dependent variables for missing data prior to application of weights. The missing data by year for all six of the dependent variables are shown in Figure 1. 

From Figure 1, there is a clear and consistent pattern of missing data for each of the dependent variables by year, which suggests that some interviewers in some states did not ask certain questions to some individuals. We coded these responses as blanks and dropped them from the analyses.

For the independent variables, missing data were less problematic. Table 1 provides a summary of the blanks, ‘do not know,’ and ‘refused’ responses for each of the independent variables (prior to weighting) under the label ‘unknown.’ 

In Table 1, the percentage of unknown is high for income. Only 2857 responses were blank, 43,345 were ‘do not know,’ and 41,816 ‘refused’.

In all cases except for gender, we merged blanks, ‘do not know,’ and ‘refused’ categories. For gender, so few were missing that we decided to impute the modal response. 

### 2.6. Inferential Methods

All dependent variables were dichotomous, and we applied complex weights so that they estimated the population of diabetics aged 45 and above for the years 2011 through 2019. The quasibinomial distribution allows for non-integer dependent variable status which occurs during complex weighting. It also estimates variance in the data not solely explained by the binomial [28]. The formula for the quasibinomial is Equation (2).

(2) $$P\left(X=k\right)=\left(\begin{array}{c} N\\ k \end{array}\right)p{\left(p+k\phi \right)}^{k-1}{\left(1-p-k\phi \right)}^{N-k}$$

In Equation (2), N is the number of weighted observations, p is the probability of dependent variable occurrence, k counts the successes, ϕ is an added variance parameter that is outside of the binomial distribution. When ϕ is zero, the above equation reverts to a standard binomial model requiring integer counts. Thus, ϕ is a critical part of the quasibinomial model.

## 3. Results

Complete results and analysis provided in this study are available online. Additional descriptive statistics, inferential statistics, and coding have also been made available [24]. 

### 3.1. Descriptive Statistics

#### 3.1.1. Dependent Variables

**Figure 2:**
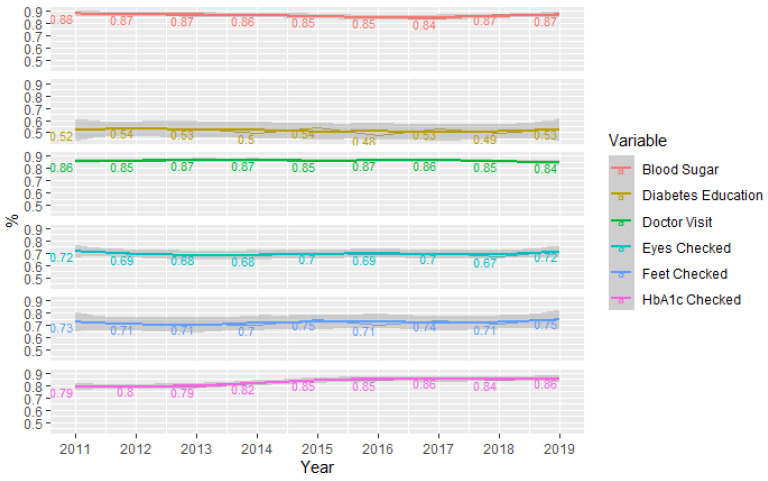
Plot of weighted diabetes management variables over time.

Time series analysis of all six dependent variables is shown in Figure 2. This figure includes plots along with locally weighted scatterplot smoothing (loess) curves. This figure depicts the population proportion estimates for each dependent variable by year.

Figure 2 highlights the stability of all variables over time. Blood sugar self-checks over time were between 84% to 88% of the population during the 9 years investigated. Similarly, HbA1c checks by doctors ranged from 79% to 86%, increasing slightly over time. Feet checks by doctors remained constant between 70 and 75%, while eye checks ranged from 67% to 72%. Diabetes education (48% in 2016 to 54% in 2011) was problematic.

#### 3.1.2. Demographics

**Figure 3:**
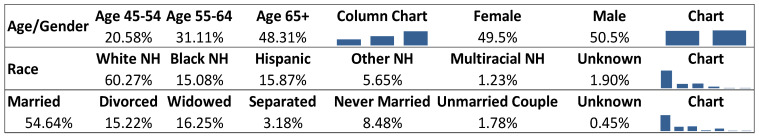
Marginal distributions for demographic variables.

Weighted demographic analysis estimated that 48.3% of the diabetic population were aged 65 or older. Most of the diabetic population were white non-Hispanic (60.9%). The proportion black non-Hispanic was 15.1% yet only 13% of the population was black [29]. The gender distribution was equally distributed, although a slight majority of the weighted population were males (50.5%). Most individual respondents were married (54.6%). We noted that these distributions remained constant over time. Figure 3 is the marginal distributions and graphs for (weighted) age, race/ethnicity, and marital status.

In Figure 3, ‘unknown’ status includes those that refused, did not know, were not sure, or had blank responses. 

#### 3.1.3. Socioeconomic Status

**Figure 4:**
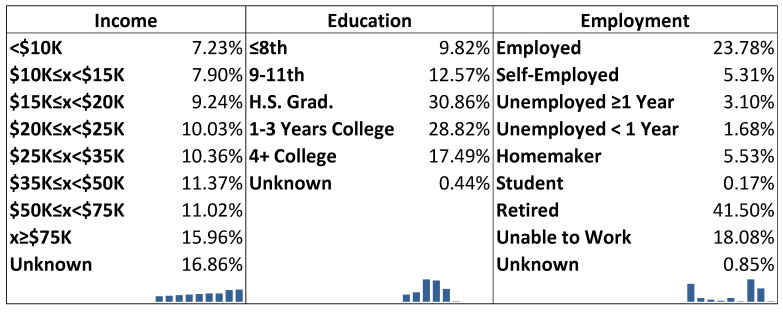
Marginal distributions for income, education, and employment.

Population estimates for income, education, and proportion suggested that most individuals with known income made at least USD 75,000 (16.0%); however, 16.9% of the income observations were not available (refused, do not know, not sure, or blank). Most of the population graduated from high school or earned a General Educational Development equivalent (30.9%). About 41.5% of the weighted sample identified as retirees. Figure 4 is the marginal distributions for income, education, and employment in proportions and graphs.

In Figure 4, the unknown category for all three groups stands for those who refused to respond, did not know, were not sure, or otherwise had blank responses. 

#### 3.1.4. Health Status

**Figure 5:**
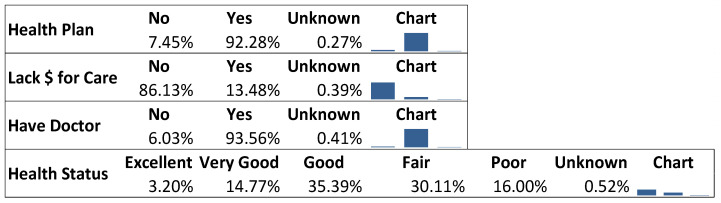
The marginal distributions for health status variables.

After application of weights, estimates showed that 92.3% of the population had health coverage under a health plan, and 93.6% had a personal doctor. About 13.5% of the population did not have the money to access healthcare due to cost constraints during the previous 12 months. The modal health status was ‘good’ (35.4%). Figure 5 is the marginal distributions for health status variables. 

### 3.2. Inferential Statistics

**Figure 6:**
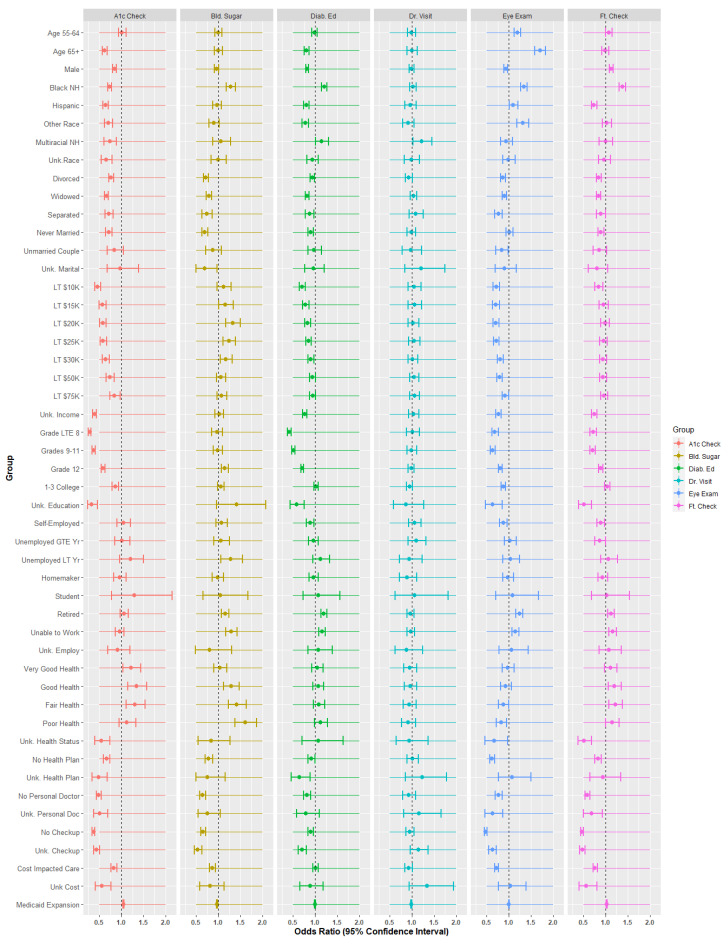
Side-by-side forest plots with vertical lines showing the 95% confidence intervals.

Quasibinomial regression models estimated predictors for the six dependent variables. The referent categories for the independent variables were as follows: age: 45 to 54, gender: female, race: white non-Hispanic, marital status: married, education: college 4+ years, employment: employed for wages, general health: excellent, health plan: yes, personal doctor: yes, checkup: yes, no money for care: no. Figure 6 and Table 2 are the forest plots and odds ratios/*p*-values for the quasibinomial models.

In Figure 5 and Table 2, ‘HbA1c’ is the dependent variable associated with physician checks of HbA1c within the last 12 months. ‘Bld. Sugar’ stands for whether the patient self-checked their blood within the last 12 months. ‘Diab Ed.’ is the response for whether patients had ever received education about their diabetes. ‘Dr. Visit’ is whether the patient visited a doctor for their diabetes within the past 12 months. ‘Eye Exam’ and ‘Ft. Check’ represent the remaining two variables, where ‘Ft.’ is an abbreviation for ‘feet.’

#### 3.2.1. Effect Sizes

Equation (3) estimated effect sizes (pseudo R^2^) for each of the six models. The effect size is 1—the model deviance divided by the null deviance, where model deviance is −2 times the log likelihood of the saturated model and null deviance is −2 times the log likelihood of the null model.

(3) $$1-\frac{-2\;\log ℒ(\hat{\beta })}{-2\;\log ℒ(\beta_{0})}$$

Effect sizes were nominal for each of the three models as shown in Table 3. Five of the models saw effect sizes less than 0.05, and the remaining variable (HbA1c checks) was just slightly better than the null model at 0.125. 

#### 3.2.2. Demographic Analysis

Age was associated with mixed results across the six dependent variables. Age 55 to 64 slightly increased the odds of having eye examinations (OR = 1.190, *p* < 0.001) and feet checks compared to age 45–54 (OR = 1.072, *p* < 0.001). Age 65 and above saw statistically increased HbA1c checks (OR = 0.623, *p* < 0.001) as well as diabetes education (OR = 0.805, *p* < 0.001) and statistically higher eye examinations (OR = 1.704, *p* < 0.001). 

Gender is associated with some diabetes management indicators. For three out of six categories, males were less likely to manage their diabetes than females. These categories include HbA1c (OR = 0.846, *p* < 0.001), diabetes education (OR = 0.820, *p* < 0.001), and eye examinations (OR = 0.932, *p* < 0.001). Males, however, were more likely to have their feet checked (OR = 1.128, *p* < 0.001). 

Race and ethnicity are associated with diabetes management indicators. Compared to white non-Hispanics, black non-Hispanics were less likely to have their HbA1c checked by doctors (OR = 0.731, *p* < 0.001) but about equally likely to have seen a doctor (no statistical difference). This group was more likely to engage in other diabetes management activities such as self-checks of blood (OR = 1.283, *p* < 0.001), diabetes education (1.205, *p* < 0.001, eye examinations (OR = 1.337, *p* < 0.001), and feet examinations (OR = 1.375, *p* < 0.001). This result might be because of provider actions based on the known relationship between race and diabetes. Hispanics, however, were much less likely than their white non-Hispanic counterparts to have their HbA1c checked (OR = 0.638, *p* < 0.001), diabetes education (OR = 0.802, *p* < 0.001), and feet checked by providers (OR = 0.736, *p* < 0.001). This group was more likely to have their eyes examined (OR = 1.103, *p* < 0.05). For the other race category, HbA1c checks (OR = 0.709, *p* < 0.001) and diabetes education (OR = 0.776, *p* < 0.001) were less likely, while eye examinations were more likely (OR = 1.314, *p* < 0.001). Multiracial individuals are less likely to have their HbA1c checked (OR = 0.736, *p* < 0.001) but more likely to have diabetes education (OR = 1.149, *p* < 0.001) and doctor visits (OR = 1.214, *p* < 0.001) compared to white non-Hispanics. Those in the ‘unknown’ category were less likely to have any diabetes management; however, only checks of HbA1c were statistically significant (OR = 0.660, *p* < 0.001). Overall, the results are suggestive of some racial and ethnic disparities in diabetes management.

Divorced individuals were much less likely than married individuals to engage in any diabetes management activities with odds ratios of 0.769, 0.719, 0.940, 0.929, 0.869, and 0.845 for HbA1c checks, blood sugar self-checks, diabetes education, doctor visit, eye exam, and feet exam, respectively. All statistically significant odds ratios for widowed, separated, never married, unmarried couple, and unknown relationship were all below 1.0, suggesting that the comparison group (married) experienced better diabetes management. Overall, marriage is associated with improved diabetes management. 

#### 3.2.3. Socioeconomic Status

Compared to those earning greater than or equal to USD 75,000, those earning less than USD 10 K were much less likely to have their HbA1c (OR = 0.466, *p* < 0.001), eyes (OR = 0.712, *p* < 0.001), or feet (OR = 0.839, *p* < 0.05) checked by physicians. They were also less likely to have diabetes education (OR = 0.709, *p* < 0.001). For all income groups less than USD 75 K, odds ratio estimates were below 1.0 for HbA1c checks, diabetes education, eye examinations, and feet checks. Those individuals in the ‘unknown’ category, which includes refusals, had strikingly low odds associated with HbA1c checks (OR = 0.393, *p* < 0.001). It is possible that many of these individuals belong in the less than USD 10,000 group. Overall, earning USD 75 K or more was associated with improved diabetes management.

Compared to those who completed four years or more of college, those with a 1st through 8th grade education had significantly lower odds of having their HbA1c (OR = 0.284, *p* < 0.001), eyes (OR = 0.685, *p* < 0.001), or feet (OR = 0.718, *p* < 0.001) checked and to have had diabetes education (OR = 0.415, *p* < 0.001). The odds ratios for those with a ninth but less than 12th grade education patterned themselves after those with first through eighth grade education. The statistically significant odds ratios for this group were 0.377, 0.507, 0.633, and 0.702 for HbA1c, diabetes education, eye checks, and feet checks, respectively. While statistically significant odds ratios for HbA1c, feet, and eye checks for those with education status of 12th grade, 1–3 years of college, or unknown were less than 1.0, odds ratios for blood sugar self-checks for these groups were statistically higher than those with four or more years of college; however, the effect size was nominal (1.148, 1.060, and 1.413 for 12th grade, 1–3 years of college, and unknown status, respectively). There appears to be a positive effect of a completed college education on diabetes management. 

Compared to those employed for wages, retirees and those unable to work were more likely to engage in self-checks of their blood sugar, (OR = 1.154 and 1.295, both *p* < 0.001), to have completed diabetes education (OR = 1.195 and 1.158, both *p* < 0.001), and to have their eyes (OR = 1.240 and 1.151, both *p* < 0.001) and feet examined (OR = 1.118 and 1.156, both *p* < 0.001). This finding may be due to opportunity cost of lost time for those working for wages. Self-employed individuals were less likely to have had diabetes education (OR = 0.885, *p* < 0.001) and feet (OR = 0.872, *p* < 0.001) or eye (OR = 0.884, *p* < 0.001) exams. 

#### 3.2.4. Health Status

There were few differences between those with ‘excellent’ health status (referent category) and those in ‘very good’ health with the exception that the latter were more likely to have HbA1c checks (OR = 1.220, *p* < 0.05). Those reporting a health status of ‘good’ were more likely to have had HbA1c checks (OR = 1.343, *p* < 0.001), blood sugar self-checks (OR = 1.290, *p* < 0.001), and feet examinations (OR = 1.199, *p* < 0.01). For those in ‘fair’ health, statistically significant odds ratios were 1.305, 1.416, 0.877, and 1.218 for HbA1c checks, blood sugar self-checks, eye exams, and feet exams, respectively. Results for those reporting poor and unknown health are similarly mixed. While health status is associated with some diabetes management variables, the effects do not appear to be directionally constant. 

Compared to individuals with health plans, those individuals with unknown health plan status were less likely to have their HbA1c checked (OR = 0.479, *p* < 0.001) and diabetes education (OR = 0.644, *p* < 0.001). Individuals with no health plan were much less likely to have diabetes management for all variables except doctor visits (no statistical difference). Odds ratios and *p*-values were 0.663 (*p* < 0.001), 0.784 (*p* < 0.001), 0.915 (*p* < 0.05), 1.013 (*p* > 0.05), 0.625 (*p* < 0.001), and 0.828 (*p* < 0.001) for HbA1c checks, blood sugar self-checks, diabetes education, doctor visit, eye checks, and feet checks, respectively. Health plan status appears to be associated with diabetes management. 

The results of the quasibinomial analysis suggest clear relationships when comparing those who do not have a doctor or unknown status to those who do. For those who do not have a personal doctor, odds ratios are below 1.0 for HbA1c checks (OR = 0.484, *p* < 0.001), blood sugar self-checks (OR = 0.646, *p* < 0.001), diabetes education (OR = 0.818, *p* < 0.001), eye exams (OR = 0.770, *p* < 0.001), and feet exams (OR = 0.588, *p* < 0.001). Only the visit to a doctor for diabetes was not statistically significant. For those in the unknown category, statistically significant odds ratios are also below 1.0 and in the areas of HbA1c checks (OR = 0.434, *p* < 0.001), self-checks of blood sugar (OR = 0.538, *p* < 0.001), diabetes education (OR = 0.705, *p* < 0.001), eye exams (OR = 0.626, *p* < 0.001), and feet exams (OR = 0.677, *p* < 0.001). The status of having a doctor appears to influence diabetes management variables. 

Compared to those who had a routine checkup by a doctor within the last year, those who did not were much less likely to engage in diabetes management, except for visiting a doctor for diabetes (no statistical difference). Odds ratios for HbA1c checks, blood sugar checks, diabetes education, doctor visit for diabetes, eye checks, and feet checks were (respectively) 0.369 (*p* < 0.001), 0.662 (*p* < 0.001), 0.895 (*p* < 0.05), 0.956 (*p* > 0.10), 0.478 (*p* < 0.001), and 0.463 (*p* < 0.001) for those who did not have a routine checkup. For those with unknown status, the odds ratios were also well below those who had visits: 0.434 (*p* < 0.001), 0.538 (*p* < 0.001), 0.705 (*p* < 0.001), 1.145 (*p* > 0.10), 0.626 (*p* < 0.001), 0.470 (*p* < 0.001), respectively. Routine checkups are associated with diabetes management variables. 

Compared to those who were able to pay for care, those who reported that they could not see a doctor within the last 12 months due to cost and those who are of unknown status were much less likely to engage in diabetes management. For those who responded that cost affected their care decisions, HbA1c checks (OR = 0.831, *p* < 0.001), self-checks of blood sugar (OR = 0.862, *p* < 0.001), eye exams (OR = 0.719, *p* < 0.001), and feet exams (OR = 0.760, *p* < 0.001) were statistically lower than those able to pay. For those cases where the true status of this question is unknown, both HbA1c (OR = 0.559, *p* < 0.001) and feet checks (OR = 0.564, *p* < 0.001) were statistically less likely. The ability to pay for care appears to affect diabetes management variables. 

#### 3.2.5. Medicaid Expansion

**Figure 7:**
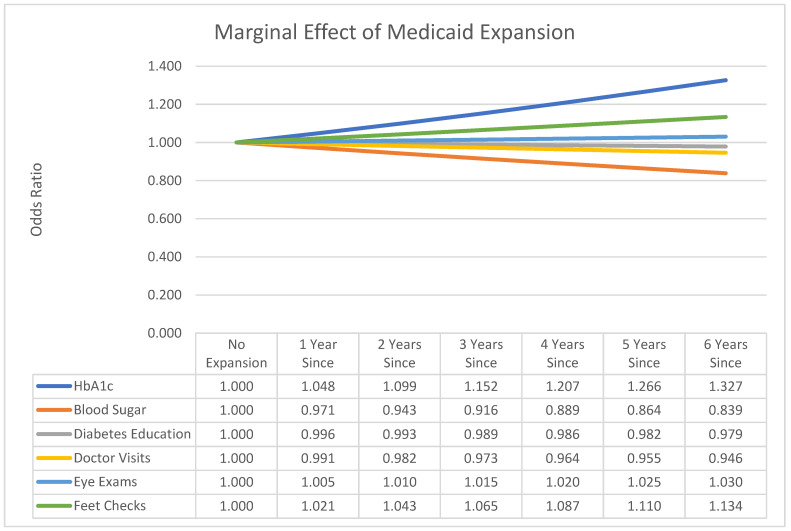
Marginal effects of Medicaid Expansion.

The Medicaid Expansion spline variable had nominal effects on diabetic management variables over time. HbA1c checks (OR = 1.048, *p* < 0.001) and feet checks (OR = 1.021, *p* < 0.001) were more likely, yet self-checks of blood sugar (OR = 0.971, *p* < 0.001) are statistically less likely. Figure 7 illustrates the time-based effects of Medicaid Expansion, the exponent of the log-odds parameter estimate. 

From Figure 7, the marginal effects of the Medicaid Expansion spline on HbA1c and feet checks show improvement over time. Diabetes education, visits to the doctor for diabetes, and self-checks of blood sugar decrease over time. 

#### 3.2.6. Sub-Model Analysis

Since this analysis focuses on explanation rather than prediction, we evaluated hierarchical subordinate models by variable group (demographics, SES, health status, and Medicaid Expansion). We evaluated all models based on the Akaike information criterion (AIC), where lower values suggest better models (Equation (4)).

(4) $$2k-2\log\hat{ℒ}$$

In Equation (3), *k* is the number of parameters in the model, so that *2k* serves as a linear penalty function, increasing the AIC. The expression, $-2\log\hat{ℒ}$, is −2 times the maximum of the likelihood function. For prediction, minimizing the AIC is equivalent to minimizing the leave-one-out cross-validation classification metric [30]. Thus, the lower values of AIC indicate better predictive models for those considered. Comparisons of these hierarchical model AICs are shown in Table 4. 

From Table 4, blood sugar checks, HbA1c checks, and feet checks favor the full model. For doctor’s visits, Medicaid Expansion is the only variable recommended by AIC (with ~zero pseudo-R^2^). For diabetes education and eye examinations, full models without Medicaid Expansion achieved the highest pseudo-R^2^ of 0.033 and 0.054, respectively. No model or sub-model is sufficiently better than the null for any variable except for HbA1c checks, where pseudo-R^2^ is 0.125. Sub-model odds ratios are directionally congruent with the full model.

## 4. Discussion

In this study, we examined changes in diabetes self-management patterns for HbA1c checks, self-checks of blood sugar, diabetes education, diabetes doctor visit, eye examinations, and feet examinations for the years 2011–2019. Improvements across time did not materialize from descriptive time series graphs, so we explored with inferential modeling based on demographic, SES, health status, and Medicaid Expansion variables. 

The demographic analysis pointed to disparities in provider checks of HbA1c. We found that providers are much less likely to check black non-Hispanic, Hispanic, other minority, multiracial, and unknown race individuals. These odds ratios (0.731, 0.638, 0.709, 0.736, 0.660, respectively) indicate that the majority white non-Hispanics are about 1.4 to 1.8 times more likely to have a provider check their HbA1c. This finding supports previous research from 2008, which found HbA1c disparities based on race [31]. Other racial and ethnic findings are of mixed direction. 

While gender differences for the six variables are mixed, they favor women. Males are less likely to have HbA1c checks, diabetes education, and eye examinations. This finding supports a previous study that found males are more likely to have poor glycemic control [32].

Compared to married individuals, all other marital status levels had odds ratios lower than zero (except for the doctor visit variable). This finding makes sense in that an additional person may remind and motivate. It is also congruent with other research that found married individuals are more likely to engage in glycemic control [33].

Aside from doctor visits (no differences) and self-checks of blood sugar, those individuals with income over USD 75,000 per year were more likely to have HbA1c, eye, and feet checks along with diabetes education. Those with the least income had the smallest odds ratios. This finding makes sense, as many low-income individuals appear to lack knowledge about proper diabetes management [34].

Some previous research has found that education status has no effect on glycemic control [35,36]. Other research suggests that college education is associated with better physical activity and glycemic control [37]. This study supports the latter finding. Specifically, college graduates were much more likely to engage in diabetes management. From a practical point of view, this finding makes sense. College graduates are likely to be educated about the risks associated with diabetes and likely to engage in prevention activities [37]. 

Further, we found that Medicaid Expansion was associated with mixed and nominal effects on diabetes management. This finding might reflect secondary effects of pay-for-performance and other factors. With the roll-back of ACA elements under the previous administration, those with chronic conditions who depend on Marketplace coverage plans were made vulnerable, as their health insurance coverage may depend on state-level efforts [5]. For this reason, our analyses might show a wearing out of the accrued positive effects that ceased after the roll-back of certain ACA provisions.

Those with a health insurance plan, those with a primary care physician, and those who had a check-up in the past year were more likely to engage in diabetes self-management. These findings align with earlier studies that identified characteristics associated with diabetes management, including having insurance/cost-related delay in care [38] and having proper communication from a provider [39].

As expected, the management of this chronic disease appeared to vary across many demographic and socioeconomic characteristics, including age, race, ethnicity, marital status, health insurance coverage, income, works status, and education. Older adults (55+) showed the greatest likelihood of engaging in diabetes management (except for doctor visits). Hispanic and ‘other race’ individuals were less likely to have their diabetes managed in most categories. Being married increased the odds for most of the diabetes self-management measures. SES analyses produced mixed results. 

Earlier studies suggest that the coverage gain from the ACA promoted access to diabetes management care with improvements in biomarkers related to the chronic disease [20]. After the ACA went into effect, millions of Americans gained health insurance coverage, particularly in states that chose to expand Medicaid. According to the Census Bureau, over 13% of Americans were uninsured in 2013 prior to when the ACA took full effect, but by 2018, 8.5% of the population was uninsured [40]. The average uninsured rate among non-elderly Americans decreased considerably from 16.8% in 2013 to 13.5% within the next year, dropping up to 10.0% in 2016 [41]. Particularly significant is the increased coverage and subsequent access to care for adults with chronic conditions in the United States [5]. Specifically, for adults with diabetes, health insurance coverage increased significantly with ACA implementation when comparing coverage rates in 2009 and 2016 [42]. Among low-income adults with diabetes, there was a drop in the uninsured proportion from 33% to 6% after the implementation of the ACA [43].

Retirees and those who could not work (disabled) were more likely than those employed for wages to have better diabetic management in terms of self-checks of blood sugar, diabetes education, eye examinations, and feet checks. Unsurprisingly, retirement has been associated with increased healthcare utilization [44].

Excellent health status was associated with higher odds for having an eye examination versus poor, fair, and unknown factor levels; however, these individuals were less likely to have their HbA1c checked. The health status variables provided no consistent, linear information about diabetes management.

Having a health plan, a primary care provider, and an annual physical are associated with higher odds of diabetes management. This is true for all variables except for doctor visits (not statistically significant). Primary care providers often set up programs specifically to address the needs of diabetics [45], and physicals are designed to address all required needs. On the other hand, individuals unable to obtain healthcare at some point during the last year due to cost are much less likely to engage in diabetes management, except for doctor’s visits (not statistically significant but less than 1.0)

Finally, Medicaid Expansion is associated with positive effects on both HbA1c and feet checks but negative effects on visits to the doctor for diabetes and self-checks of blood sugar. The effect sizes are small, as illustrated in Figure 7.

## 5. Conclusions

Our findings do not show a significant effect of Medicaid Expansion on diabetes management, although we do document the estimated effect of demographic and SES factors. The improvements in HbA1c might reflect measures often included in pay-for-performance (P4P) programs, many of which include HbA1c as opposed to the other self-management behaviors examined in this study. A review of P4P programs in the literature found that studies on diabetes included HbA1c as the most cited program measured. Although P4P increases the delivery of services included in quality measure sets, other measures may receive little or no attention. This phenomenon was observed in the United Kingdom where the Quality and Outcomes Framework led to improvements in measures included in the incentive program while the quality of care for other conditions suffered [46]. 

Despite expanded insurance coverage, barriers persist. For example, accounting for 25 cents of every dollar spent by individuals with diabetes on pharmaceuticals, the cost of testing supplies has outpaced inflation. Likewise, insurance expansion alone may be insufficient to increase screening for retinopathy. Distance to screening, financial concerns, and lack of time are barriers unaddressed by the ACA. Tele-retinopathy screening has the potential to address these challenges though adoption of this innovation could be similarly uneven. Another trend influencing patient behavior is the rise of high deductible plans, which keep premiums low by discouraging unnecessary utilization. In response, some patients have delayed all care, including services needed to control chronic diseases and prevent complications. 

Previous findings suggest that expanding Medicaid coverage may provide improved access to health services that support chronic disease management [47]. Another recent study found that Medicaid Expansion positively impacted self-reported diabetes management [15]. Additionally, Medicaid Expansion may be associated with lower rates of disruptions in health insurance coverage [48]. However, over 2 million Americans residing in states that did not expand Medicaid unfortunately fall into a coverage gap [41]. These individuals, who tend to be from southern states with the largest populations of those in this coverage gap, do not qualify for Medicaid in their states but cannot afford Marketplace premium tax credits.

There are several limitations to this study. First, the BRFSS excludes individuals without landlines or cellular phones, and those residing in institutions [49]. Second, BRFSS data are self-reported so there is an inherent bias over validated medical histories. Third, BRFSS data, while generally valid and reliable [22], may be misinterpreted. 

In summary, we found that Medicaid Expansion had mixed effects on the dependent variables. This finding might derive from the fact that diabetes diagnoses increased after the expansion and those patients had yet to begin diabetes self-management. Since Medicaid Expansion likely increased use and access for low-income diabetics [50], the population then likely also shifted. While another small study suggested that Medicaid expansion had effects on self-reported access, health status, and diabetes management [15], we find little effect size substantiation in this larger study using linear splines for state-based implementation. We recommend further study to evaluate if the population estimates associated with diabetes management remain stable over time and become explainable based on Medicaid Expansion.

## Figures and Tables

**Table 1:** Independent variables.

	Type	# of Factor Levels	Unknown	% Unknown
Age	Categorical	3	-	0.00%
Race	Categorical	6	9701	1.90%
Gender	Categorical	3	207	0.04%
Marital Status	Categorical	7	2612	0.51%
Income	Categorical	9	88,018	17.22%
Education	Categorical	6	2169	0.42%
Work Status	Categorical	9	3704	0.72%
Health Plan	Categorical	3	1452	0.28%
Personal Doctor	Categorical	3	1826	0.36%
Annual Checkup	Categorical	3	8075	1.58%
Cost Affected Care	Categorical	3	1890	0.37%
Health Status	Categorical	6	2252	0.44%
Medicaid Expansion	Quantitative	N/A	-	0.00%

**Table 2:** Odds ratios and associated *p*-values for the quasibinomial models.

	HbA1c		Bld. Sugar		Diab. Ed.		Dr. Visit		Eye Exam		Ft. Check	
Intercept	26.418	***	4.808	***	1.915	***	6.731	***	3.135	***	3.055	***
Age 55–64	1.015		0.998		0.985		0.986		1.190	***	1.072	*
Age 65+	0.623	***	1.001		0.805	***	1.000		1.704	***	0.992	
Male	0.846	***	0.961		0.820	***	0.991		0.932	***	1.128	***
Black	0.731	***	1.283	***	1.205	***	1.021		1.337	***	1.375	***
Hispanic	0.638	***	0.968		0.802	***	0.961		1.103	*	0.736	***
Other	0.709	***	0.899		0.776	***	0.912		1.314	***	1.024	
Multirace	0.736	**	1.060		1.149	*	1.214	*	0.936		0.995	
Unk. Race	0.660	***	0.995		0.938		0.985		0.991		0.961	
Divorced	0.769	***	0.719	***	0.940	*	0.929	^+^	0.869	***	0.845	***
Widowed	0.662	***	0.790	***	0.821	***	1.036		0.896	***	0.844	***
Separated	0.716	***	0.740	***	0.872	*	1.083		0.764	***	0.894	^+^
Nvr. Married	0.711	***	0.692	***	0.899	**	0.987		1.016		0.895	**
Unm. Couple	0.840		0.880		0.979		0.977		0.835	*	0.859	^+^
Unk. Relationship	0.974		0.695	*	0.964		1.211		0.900		0.802	
<$10 K	0.466	***	1.123		0.709	***	1.047		0.712	***	0.839	**
<$15 K	0.571	***	1.162	*	0.784	***	1.055		0.707	***	0.952	
<$20 K	0.579	***	1.323	***	0.830	***	1.026		0.703	***	0.986	
<$25 K	0.588	***	1.241	***	0.849	***	1.046		0.712	***	0.950	
<$30 K	0.643	***	1.174	**	0.902	**	1.017		0.803	***	0.939	
<$50 K	0.744	***	1.061		0.939	^+^	1.054		0.789	***	0.943	
<$75 K	0.845	*	1.077		0.945		1.057		0.919	*	0.969	
Unk. $	0.393	***	1.018		0.765	***	1.040		0.762	***	0.739	***
1th–8th	0.284	***	0.970		0.415	***	1.016		0.685	***	0.718	***
9th–12th	0.377	***	0.992		0.507	***	0.992		0.633	***	0.702	***
12th	0.586	***	1.148	***	0.711	***	0.982		0.807	***	0.889	***
1–3 College	0.864	***	1.060	^+^	1.027		0.946		0.872	***	1.036	
Unk. Ed.	0.329	***	1.413	^+^	0.577	***	0.861		0.637	**	0.512	***
Self-Employed	1.046		1.070		0.885	**	1.059		0.872	**	0.884	*
No Work 1 yr.+	1.008		1.064		0.956		1.096		1.023		0.864	*
No Work <1 yr.	1.204	^+^	1.279	*	1.124		0.940		1.036		1.066	
Homemaker	0.962		0.983		0.959		0.894		0.978		0.929	
Student	1.293		1.051		1.070		1.060		1.084		1.020	
Retired	1.058		1.154	***	1.195	***	0.964		1.240	***	1.118	***
Cannot Work	0.958		1.295	***	1.158	***	0.969		1.151	***	1.156	***
Unk. Work	0.913		0.798		1.077		0.875		1.061		1.074	
Very Good	1.220	*	1.036		1.044		0.946		0.977		1.111	
Good	1.343	***	1.290	***	1.067		0.957		0.929		1.199	**
Fair	1.305	**	1.416	***	1.086		0.941		0.877	*	1.218	**
Poor	1.124		1.608	***	1.125	^+^	0.913		0.827	**	1.145	*
Unk. Health	0.542	***	0.836		1.074		0.936		0.672	*	0.509	***
No Hlth Plan	0.663	***	0.784	***	0.915	*	1.013		0.625	***	0.828	***
Unk. Hlth Plan	0.479	***	0.757		0.644	**	1.231		1.075		0.932	
No Doctor	0.484	***	0.646	***	0.818	***	0.927		0.770	***	0.588	***
Unk. Doctor	0.512	***	0.760	^+^	0.796		1.160		0.632	**	0.677	*
No Checkup	0.369	***	0.662	***	0.895	**	0.956		0.478	***	0.463	***
Unk. Checkup	0.434	***	0.538	***	0.705	***	1.145		0.626	***	0.470	***
Cost Affected	0.831	***	0.862	***	1.013		0.924		0.719	***	0.760	***
Unk. Cost	0.559	***	0.816		0.885		1.345		1.033		0.564	**
Medicaid Expansion	1.048	***	0.971	***	0.996		0.991		1.005		1.021	***

*** *p* < 0.001, ** *p* < 0.01, * *p* < 0.05, ^+^ *p* < 0.10.

**Table 3:** Pseudo-R^2^.

Variable	Effect Size
Blood Sugar	0.020
Doctor Visits	0.001
HbA1c Checks	0.125
Feet Checks	0.042
Diabetes Education	0.033
Eye Checks	0.054

**Table 4:** Akaike information criterion for sub-models.

Group	Blood Sugar	Doctor Visit	HbA1c Checks	Feet Checks	Education	Eye Exam
Demographics	158,197	161,170	171,814	226,600	269,802	237,409
SES	158,199	161,273	164,780	225,991	265,841	237,076
Health	156,833	161,142	169,671	223,338	271,800	234,101
Medicaid Expansion	158,731	**161,096**	178,049	229,191	272,790	242,692
Demographics + SES	157,615	161,337	162,816	224,604	264,410	234,478
SES + Health	156,547	161,318	158,972	221,271	265,570	231,622
Demographics + Health	156,390	161,222	163,559	221,366	269,090	231,463
Demographics + Medicaid Expansion	158,148	161,172	171,410	226,472	269,799	237,341
SES + Medicaid Expansion	158,143	161,274	164,568	225,918	265,843	237,056
Health + Medicaid Expansion	156,752	161,165	167,452	223,195	271,241	232,153
Demographics + SES + Health	156,045	161,386	156,825	220,074	**264,184**	**230,006**
Demographics + SES + Medicaid Expansion	157,576	161,338	162,594	224,520	264,415	234,461
Demographics + Health + Medicaid Expansion	156,322	161,222	163,315	221,311	269,094	231,455
SES + Health + Medicaid Expansion	156,466	161,318	158,839	221,241	265,571	231,626
Full Model	**155,986**	161,387	**156,684**	**220,033**	264,189	230,010

**Bolded:** minimum AIC.

## Data Availability

Data are available from the CDC (https://www.cdc.gov/brfss/index.html, accessed on 20 July 2021).
